# Should we investigate mitochondrial disorders in progressive adult-onset undetermined ataxias?

**DOI:** 10.1186/s40673-020-00122-0

**Published:** 2020-08-24

**Authors:** José Luiz Pedroso, Wladimir Bocca Vieira de Rezende Pinto, Orlando Graziani Povoas Barsottini, Acary Souza Bulle Oliveira

**Affiliations:** 1 Ataxia Unit, Department of Neurology and Neurosurgery, Universidade Federal de São Paulo (UNIFESP), Pedro de Toledo Street, 650. ZIP CODE: 04039-002. Vila Clementino, São Paulo, SP Brazil; 2 Division of Neuromuscular Diseases, Department of Neurology and Neurosurgery, Universidade Federal de São Paulo (UNIFESP), São Paulo, SP Brazil

**Keywords:** Ataxia, MERRF syndrome, Mitochondrial diseases, Cerebellar ataxia

## Abstract

**Background:**

Despite the broad development of next-generation sequencing approaches recently, such as whole-exome sequencing, diagnostic workup of adult-onset progressive cerebellar ataxias without remarkable family history and with negative genetic panel testing for SCAs remains a complex and expensive clinical challenge.

**Case presentation:**

In this article, we report a Brazilian man with adult-onset slowly progressive pure cerebellar ataxia, which developed neuropathy and hearing loss after fifteen years of ataxia onset, in which a primary mitochondrial DNA defect (MERRF syndrome - myoclonus epilepsy with ragged-red fibers) was confirmed through muscle biopsy evaluation and whole-exome sequencing.

**Conclusions:**

Mitochondrial disorders are a clinically and genetically complex and heterogenous group of metabolic diseases, resulting from pathogenic variants in the mitochondrial DNA or nuclear DNA. In our case, a correlation with histopathological changes identified on muscle biopsy helped to clarify the definitive diagnosis. Moreover, in neurodegenerative and neurogenetic disorders, some symptoms may be evinced later during disease course. We suggest that late-onset and adult pure undetermined ataxias should be considered and investigated for mitochondrial disorders, particularly MERRF syndrome and other primary mitochondrial DNA defects, together with other more commonly known nuclear genes.

Dear Editor,

According to its etiological basis, hereditary ataxias are classified into six major groups: autosomal dominant spinocerebellar ataxias (SCA), autosomal recessive, congenital, mitochondrial, episodic and X-linked cerebellar ataxias [1, 2]. Despite the broad development of next-generation sequencing approaches recently, such as whole-exome sequencing (WES), diagnostic workup of adult-onset progressive cerebellar ataxias without remarkable family history and with negative genetic panel testing for SCAs remains a complex and expensive clinical challenge [1–3]. In this article, we report a Brazilian man with adult-onset slowly progressive pure cerebellar ataxia, which developed neuropathy and hearing loss after fifteen years of ataxia onset, in which a primary mitochondrial DNA (mtDNA) defect was confirmed through muscle biopsy evaluation and WES.

A 66-year-old man presented with slow progressive ataxia that started 20 years before. When he was 46-year-old, mild loss of balance started. Parents were non-consanguineous. Family history was unremarkable. Examination disclosed moderate to severe cerebellar ataxia, dysmetria and dysarthria. An extensive investigation, during the first 15 years of disease onset, resulted negative. SCA genetic panel, Friedreich ataxia, autoimmune disorders (GAD, thyroid antibodies, celiac disease), paraneoplastic panel, vitamin levels, sensory and motor neuroconduction studies and needle electromyography (EMG) were normal. Basic metabolic work-up with plasma lactate, ammonia and lactate/pyruvate ratio was unremarkable. Brain magnetic resonance imaging (MRI) disclosed global cerebellar atrophy (Fig. 1). Gene panel testing for cerebellar ataxias including *SYNE1*, *SPG7* and *SACS* genes resulted negative. A first WES testing was inconclusive.

**Fig. 1:**
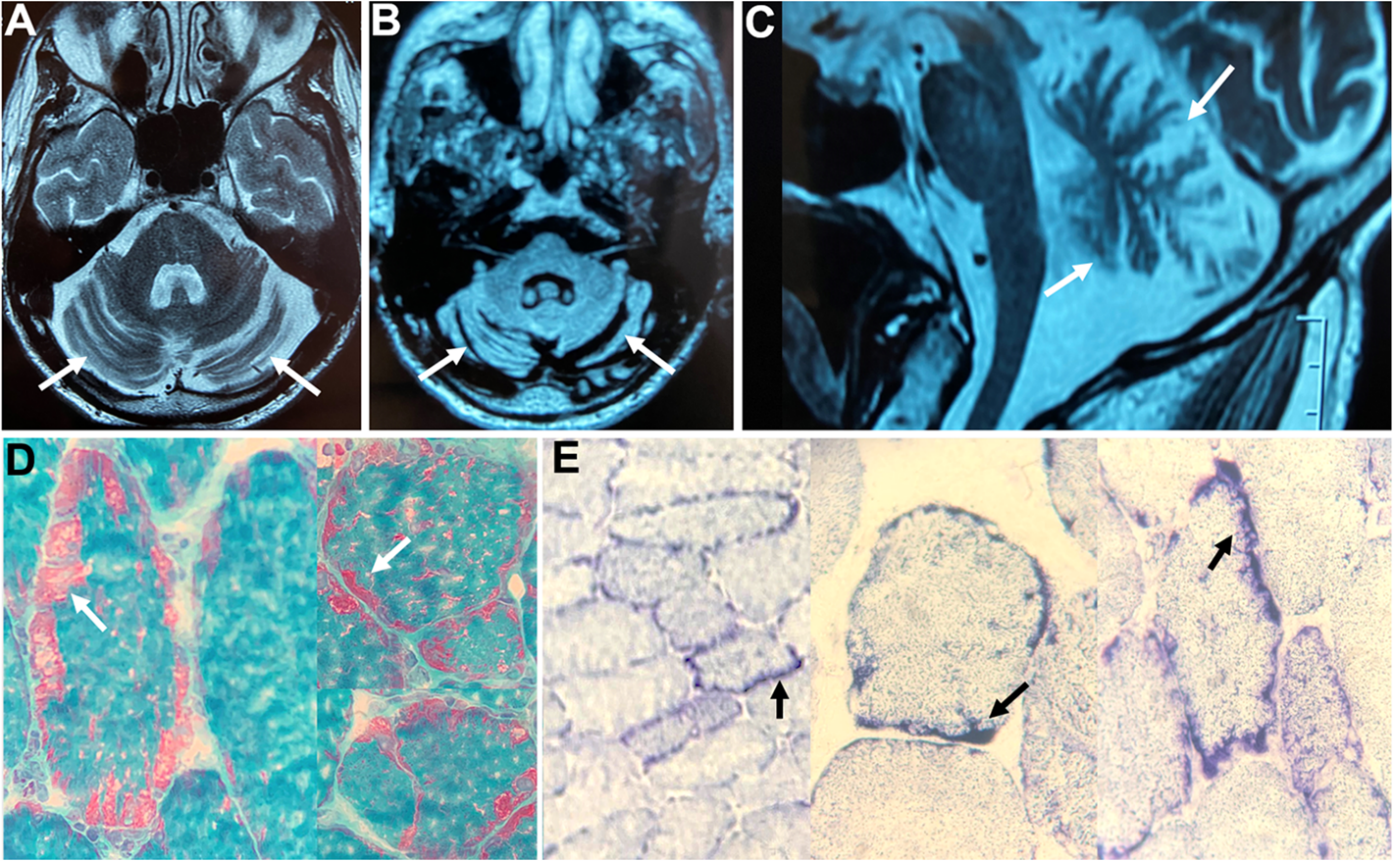
(**a**) Axial T2-weighted, (**b**) axial FLAIR and (**c**) sagittal T2-weighted brain MR imaging showing global cerebellar atrophy (white arrows) with normal brainstem volume. Muscle biopsy disclosed increased subsarcolemmal mitochondrial proliferation with typical ragged-red fibers (RRF) in modified Gömöri Trichrome stain (**d**; white arrows) and ragged-blue fibers in modified succinate-dehydrogenase (SDH) reaction (E; black arrows)

After fifteen years, the patient developed mild bilateral sensorineural hearing loss. Examination showed cerebellar ataxia, decreased deep tendon reflexes and distal weakness in lower limbs (Supplemental Video). Neuroconduction studies disclosed axonal sensorimotor polyneuropathy. The association of cerebellar ataxia with sensorineural deafness and axonal neuropathy leads to clinical suspicion of primary mtDNA defects. A second WES testing including coverage of the entire mtDNA was requested, and the A8344G pathogenic variant in the *MT-TK* gene was identified. Muscle biopsy disclosed increased subsarcolemmal mitochondrial proliferation with ragged-red fibers (Fig. 1). MERRF (myoclonus epilepsy with ragged-red fibers) genetic spectrum presenting with pure adult-onset cerebellar ataxia was diagnosed.

Mitochondrial disorders are a clinically and genetically complex and heterogenous group of metabolic diseases, resulting from pathogenic variants in the mtDNA or nuclear DNA [4, 5]. Furthermore, mitochondrial disorders are characterized by systemic involvement, and common nervous system symptoms include seizures, cerebellar ataxia, neuropathy, encephalopathy, stroke-like episodes, visual loss and deafness [6]. Although most forms start in childhood, adult-onset is not uncommon, especially when associated with other multisystemic and neurological findings [4, 5]. It is quite unusual that mitochondrial disorders present with late-onset progressive ataxia as an isolated syndrome [7]. The most common syndromic mitochondrial diseases presenting with cerebellar ataxia as a cardinal sign include: (i) Kearns-Sayre syndrome; (ii) NARP (neuropathy, ataxia and retinitis pigmentosa) syndrome; (iii) *POLG* gene spectrum disorders, highlighting the mitochondrial recessive ataxia syndrome (MIRAS), including SANDO (sensory ataxic neuropathy, dysarthria and ophthalmoplegia) and Mitochondrial Spinocerebellar ataxia with epilepsy syndrome (MSCAE); (iv) MELAS syndrome (mitochondrial encephalopathy, lactic acidosis and stroke-like episodes); and (v) primary coenzyme Q10 deficiency [4–7].

Although MERRF may present with ataxia, typical clinical features include myoclonus, seizures, myopathy and variable degrees of cognitive impairment, visual loss, deafness and neuropathy [7, 8]. Hardly ever MERRF may present with pure ataxia, particularly in adult-onset phenotypes. In a large series of patients with progressive ataxia, 9% of the patients presented with muscle biopsy suggestive of mitochondrial disorder [7, 8]. In our case, the rise of hearing loss and neuropathy guided for a mitochondrial disorder. Next-generation sequencing associated with muscle biopsy confirmed the diagnosis of MERRF.

This is a very instructive case of a patient with late-onset undetermined pure cerebellar ataxia that only lately developed hearing loss and neuropathy, and was diagnosed through genetics and muscle biopsy within the expanding clinical spectrum of MERRF syndrome. Despite the rare descriptions of mtDNA point mutations leading to pure cerebellar ataxia phenotypes, late-onset mtDNA defect cases most commonly remain undiagnosed [2, 8, 9]. With the greater availability and current use of WES in clinical practice, unusual genetic causes of ataxias may be identified and need to be properly correlated with clinical findings. However, on the other hand, for atypical late-onset pure presentations, interpretation of genetic findings on WES may become sometimes a problem rather than diagnostic solving [10–12].

In our case, a correlation with histopathological changes identified on muscle biopsy helped to clarify the definitive diagnosis. Moreover, in neurodegenerative and neurogenetic disorders, some symptoms may be evinced later during disease course. In conclusion, we suggest that late-onset and adult pure undetermined ataxias should be considered and investigated for mitochondrial disorders, particularly MERRF syndrome and other primary mtDNA defects, together with other more commonly known nuclear genes. Neuropathy and hearing loss during disease course may also aid in the proper evaluation of a suspected mitochondrial disorder.

## Supplementary information

**Additional file 1 Supplemental video**. Patient with MERRF (myoclonus epilepsy with ragged-red fibers) presenting with gait ataxia, lower limb weakness related to neuropathy and dysmetria.

## Abbreviations

SCA: Spinocerebellar ataxias
WES: Whole-exome sequencing
mtDNA: Mitochondrial DNA
EMG: Electromyography
MRI: Magnetic resonance imaging
MERRF: Myoclonus epilepsy with ragged-red fibers
NARP: Neuropathy, ataxia and retinitis pigmentosa
MIRAS: Mitochondrial recessive ataxia syndrome
SANDO: Sensory ataxic neuropathy, dysarthria and ophthalmoplegia
MELAS: Mitochondrial encephalopathy, lactic acidosis and stroke-like episodes
